# Quantification of Detergents Complexed with Membrane Proteins

**DOI:** 10.1038/srep41751

**Published:** 2017-02-08

**Authors:** Vincent Chaptal, Frédéric Delolme, Arnaud Kilburg, Sandrine Magnard, Cédric Montigny, Martin Picard, Charlène Prier, Luca Monticelli, Olivier Bornert, Morgane Agez, Stéphanie Ravaud, Cédric Orelle, Renaud Wagner, Anass Jawhari, Isabelle Broutin, Eva Pebay-Peyroula, Jean-Michel Jault, H. Ronald Kaback, Marc le Maire, Pierre Falson

**Affiliations:** 1 Laboratory of Molecular Microbiology and Structural Biochemistry, CNRS UMR 5086, IBCP, Lyon 69367 France; 2 Protein Science Facility, UMS 3444, IBCP, Lyon 69367, France; 3 Institute for Integrative Biology of the Cell (I2BC), CEA, CNRS, University of Paris-Sud, 91198 Gif-sur-Yvette, France; 4 Laboratoire de Biologie Physico-Chimique des Protéines Membranaires, CNRS UMR 7099, 75005 Paris, France; 5 Institut de Recherche de l’Ecole de Biotechnologie de Strasbourg, University of Strasbourg-CNRS, Illkirch, France; 6 CALIXAR, 60 Avenue Rockefeller - 69008 Lyon, France; 7 Institut de Biologie Structurale (IBS), University of Grenoble Alpes, CEA, CNRS, 38044 Grenoble, France; 8 Laboratoire de Cristallographie et RMN Biologiques, CNRS UMR 8015, 75006 Paris, France; 9 Department of Physiology, David Geffen School of Medicine, UCLA, Los Angeles, CA 90095 USA

## Abstract

Most membrane proteins studies require the use of detergents, but because of the lack of a general, accurate and rapid method to quantify them, many uncertainties remain that hamper proper functional and structural data analyses. To solve this problem, we propose a method based on matrix-assisted laser desorption/ionization mass spectrometry (MALDI-TOF MS) that allows quantification of pure or mixed detergents in complex with membrane proteins. We validated the method with a wide variety of detergents and membrane proteins. We automated the process, thereby allowing routine quantification for a broad spectrum of usage. As a first illustration, we show how to obtain information of the amount of detergent in complex with a membrane protein, essential for liposome or nanodiscs reconstitutions. Thanks to the method, we also show how to reliably and easily estimate the detergent corona diameter and select the smallest size, critical for favoring protein-protein contacts and triggering/promoting membrane protein crystallization, and to visualize the detergent belt for Cryo-EM studies.

Detergents play a major role in handling membrane proteins. They are indispensable tools for extracting membrane proteins from the membrane and maintaining them in a soluble and active state for further study. A typical problem that arises during extraction, purification and crystallization is difficulty in controlling detergent concentration and composition, especially when detergent mixtures are used. Indeed, little is known about the fundamentals of detergent behavior around membrane proteins. Pioneering and important studies were conducted with radioactive detergents, which allowed estimation of the ratio of detergent to hydrophobic area^1^,^2^. Moreover, use of radiolabeled detergents led to crystallization of the bovine ADP/ATP carrier and sarcoplasmic reticulum Ca^2+^-ATPase, which was found to be highly dependent on the detergent:membrane protein ratio^3^,^4^. However, only a few types of radioactive detergents are available, making this approach generally untenable. Other methods developed to measure detergent concentrations include: (i) colorimetric assays to estimate the sugar moiety for specific detergents^5^,^6^; (ii) Fourier transform Infrared spectroscopy^7^; (iii) plain thin layer chromatography coupled with densitometric quantification, or more recently coupled with laser densitometry^8^,^9^; drop-shape based quantification^10^; (iv) liquid chromatography/ESI-MS^11^; (v) size-exclusion chromatography coupled with multi-angle laser light scattering^12^,^13^ and analytical ultracentrifugation^14^. Although useful, these methods are laborious, difficult to implement routinely, limited to a given type of detergent or inapplicable to detergent mixtures. Moreover, hundreds of detergents are now commercially available, rendering these methods generally impractical.

We now describe a novel and straightforward detergent quantification method based on MALDI-TOF MS that is applicable to any type of detergent or membrane protein. The new method is efficient, less cumbersome, and can be applied routinely to a wide variety of membrane proteins, thereby allowing exploration of detergent behavior under different conditions. The method provides a rapid means of assaying the amount of bound detergent surrounding a membrane protein and to estimate the size of the detergent corona.

## Results

### Detergent quantification by MALDI-TOF MS

**Figure 1:**
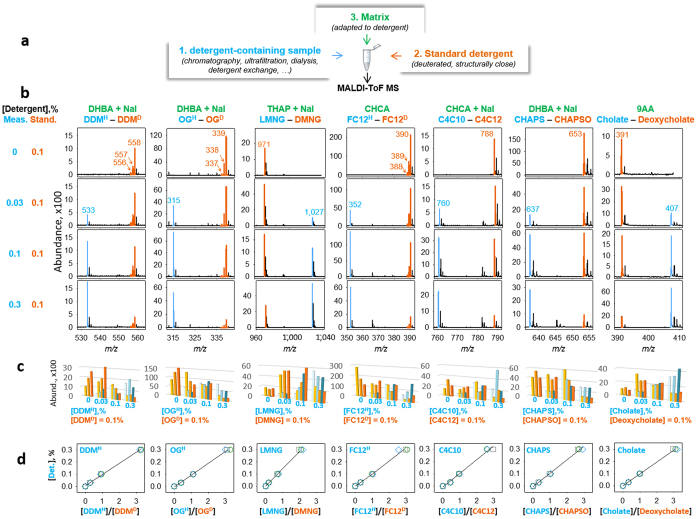
Detergent quantification by MALDI-TOF MS. (**a**) Principle of the method. Components are colored in blue, orange and green for the measured detergents, standard detergents and the desorption-helper matrix, respectively. (**b**) Detergent desorption. Detergent m/z are indicated, plus the mass of Na when NaI was added to the mixture. The different combinations of assayed and standard detergents are displayed from top to bottom, with respect to the ratios indicated on the left of the panel. (**c**) Abundance distribution from 3 independent experiments displayed in light to dark colors, blue-type for measured detergents and orange-type for standards. (**d**) Calibration curves, plotted as the amount of measured detergent (%, w/v) with respect to assayed/standard detergent abundance ratios. Circle, square and diamond correspond to 3 experiments, fitted with a linear regression.

We developed a method based by MALDI-TOF MS to quantify detergents (Fig. 1), a concept inspired by methods previously used for the quantification of proteins and peptides^15^ or of arylphosphoniums^16^. A sample containing a detergent or a mixture of detergents to be quantified is mixed with a known amount of another detergent used as internal standard (panel a). This standard is chosen to have a molecular weight (MW) distinct from the detergent in the sample but similar physicochemical properties to desorb similarly. It is then straightforward to use a simple calibration curve done in parallel to calculate the detergent concentration within the sample.

We tested the method with various detergents used for structural and functional biology, including (Fig. 1b) sugar derivatives such as n-dodecyl-β-D-maltoside (DDM), n-octyl-β-D-glucoside (OG) and lauryl maltose neopentyl glycol (LMNG)^17^, ionic detergents such as Fos-Choline 12 (FC12) and 3-[(3-cholamidopropyl)-dimethylammonio]-1-propane sulfonate (CHAPS), anionic detergents such as decyl tris[carboxymethyl]-monoalkoxy-trihydroxycalix[4]arene (C4C10)^18^, and bile-type detergents such as CHAPS and cholate. When available, standards were deuterated, e.g. for DDM, OG and FC12. In this case, we took into account the isotopic distribution of the deuterated molecules to improve the fits (Supplementary Figure 1 and Supplementary Information). Standards were also chosen structurally close for the other detergents: decyl MNG (DMNG) for LMNG, C4C12 for C4C10, 2-hydroxy CHAPS (CHAPSO) for CHAPS and deoxycholate for cholate. Importantly, the desorption-helper matrices were chosen according to their ability to obtain a homogeneous deposit and to display an efficient desorption and ionization, particularly critical for automatization. For each couple of detergents, solutions contained 0.1% (w/v) standard and 0, 0.03, 0.1 and 0.3% of measured detergent, resulting in ratios of 0, 0.3, 1 and 3, were mixed with their optimized matrix and then submitted to MS (see Methods). For further high-throughput analysis, we automated the MALDI-TOF MS acquisition process, for treating up to 100 samples in a row.

Note that deuterated internal standards are optimal as they desorb identically than their protonated detergents, but they are very expensive. To reduce this cost, they can easily be replaced by closely related molecules for routine use, e.g. Undecyl β-D Maltoside (UDM) to quantify DDM (Supplementary Figure 3), Nonyl Glucoside to quantify OG, FC14 to quantify FC12, etc.

### Limit of detection and quantification

**Figure 2:**
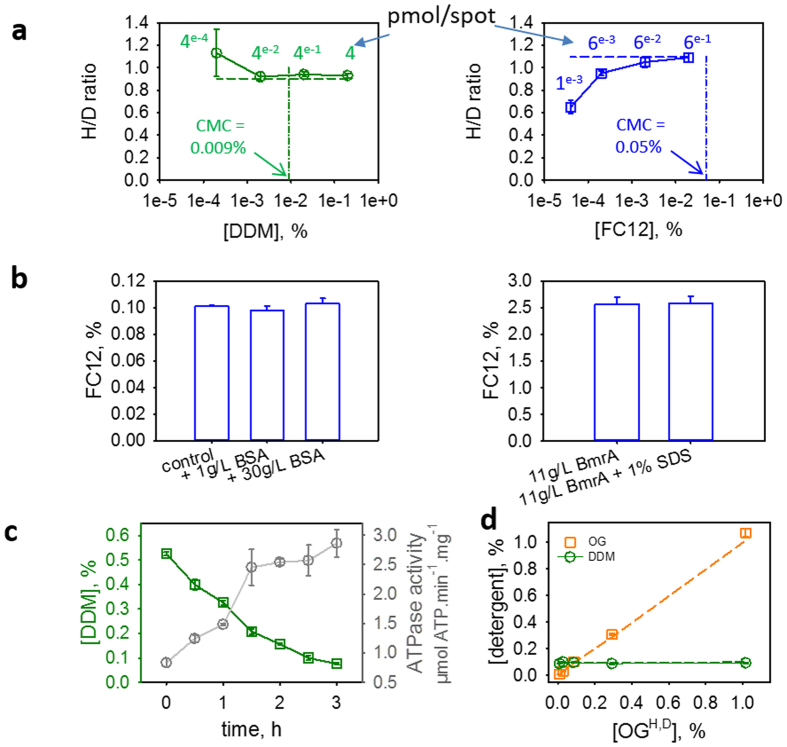
Validation of the detergent quantification by MALDI-TOF MS. (**a**) Detection limit. Detergent solutions prepared at 0.2 and 0.02% respectively, at a H/D ratio of 0.9 for DDM and 1.1 for FC12 (dashed lines), were diluted as indicated and then quantified. (**b**) The total amount of FC12 is quantified. Left, FC12 added at 0.1% in water quantified in the absence or presence of 1 or 30 g/L BSA; right: FC12 quantified in a concentrated solution of the purified membrane protein BmrA in absence or presence of 1% SDS. (**c**) DDM removal by Biobeads during protein reconstitution into lipids. DDM was quantified with time, probing the ATPase activity of the protein. (**d**) Mixing two detergents has no impact on their quantification. OG^H^ + OG^D^ (orange) mixed together at the indicated concentrations were added to 0.1% of DDM^H^ + DDM^D^ (green) and then quantified. Quantifications were done in triplicate on the same experiment.

We evaluated the limit of detection (LOD) and quantification (LOQ) of the method with DDM and FC12, by measuring the H/D ratios after sequential dilutions, until the signal to noise ratio prevented to reach the limit values of 10 (LOQ) or 3 (LOD) (Fig. 2a). Starting from 0.2% DDM (39.2 mM, left panel) and 0.02% FC12 (5.6 mM, right panel) we accurately quantified both detergents after a 100-fold dilution, and still detected them after a 10 times further dilution. Ultimately, these two detergents can be detected to values 10 and 100 times below their critical micellar concentration (CMC). The 10-fold lower concentration for FC12 than for DDM was set to prevent saturation of the detector because of the better desorption-ionization process for FC12.

### The total amount of detergent is detected

Since membrane proteins bind detergents, one critical question is whether the method detects all the detergent in a sample or only the free fraction, which would introduce a bias in the measurement. For this matter, we use MALDI-MS that is known to bring sufficient ionization energy to disrupt non-covalent interactions. We started with bovine serum albumin (BSA), known to bind 7 to 9 mol of aliphatic detergents per mol of BSA^19^. Mixing 0.1% FC12 with 1 or 30 g/L BSA, we then quantified the same FC12 concentration in both samples, 0.098% and 0.103%, respectively (Fig. 2b, left panel). As BSA should complex 0.004% and 0.13% (w/v) of the detergent in these conditions - which means that the total amount of FC12 should be complexed by BSA at 30 g/L- these results show that all the FC12 is detected in the sample irrespective of the presence of the protein. Further, we measured the total amount of FC12 in a sample of purified and concentrated membrane protein, the ABC transporter BmrA^20^, in the presence or the absence of 1% SDS (Sodium Dodecyl Sulfate). SDS would compete with FC12, and to some extent release FC12 surrounding BmrA. As shown in Fig. 2b (right panel), we detected the same amount of FC12 irrespective of the presence of SDS, confirming that the interaction of membrane proteins with detergents is sufficiently weak to release all the detergent bound upon laser desorption.

### Membrane protein reconstitution in liposomes

Reconstitution in liposomes or nanodiscs is commonly employed to re-insert membrane proteins in a lipid environment, which is often critical to study their function^21^,^22^. For this purpose, removal of detergent is conveniently achieved by using biobeads^23^,^24^. We performed a liposome reconstitution of BmrA^25^ in the presence of biobeads, and in parallel, we used our method to quantify the remaining DDM. In the presence of biobeads, the amount of DDM decreased with time (Fig. 2c), with most of the DDM removed after 3 h to a value close to its CMC. As expected, the ATPase activity of the transporter increased to its maximum during the reconstitution process.

### Detergent quantification in a mixture

As introduced, most of the methods are not suitable for detergent quantification in mixtures. In order to assess the capacity of our method to do it, we quantified known amounts of a mixture of OG and DDM, two of the most widely used detergents in membrane proteins handlings (Fig. 2d). We kept the DDM concentration constant at 0.1%, and varied the OG concentration from 0.01 to 1%. Both detergents were quantified in the range of concentrations sampled. However, at the lower limit of the experiment, we reached the limits of detection of such a detergent mix. Despite this technical limitation, the quantification of DDM remained remarkably accurate within the range of concentrations tested. Reciprocally, we quantified accurately low OG concentrations in the presence of a constant amount of DDM.

### Validation of the method with the use of radiolabeled detergent

**Figure 3:**
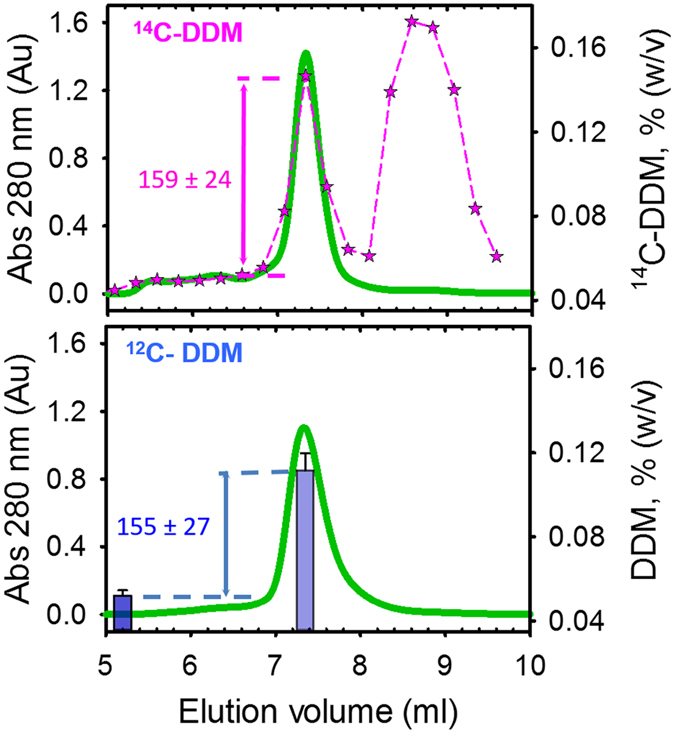
Compared quantification of DDM bound to the SERCA1a Ca^2+^-ATPase. The protein was extracted with radiolabeled (upper panel) or cold (lower panel) DDM and then submitted to two successive SEC. Green traces correspond to the absorbance at 280 nm. ^14^C-DDM was quantified by liquid scintillation (pink stars) and ^12^C-DDM by MALDI-TOF MS (blue bars). Quantifications were done in triplicate on the same experiment.

We compared the present method with the use of ^14^C-DDM as a reporter of detergent binding around membrane proteins, mainly developed with SERCA1a, the Sarco-endoplasmic Reticulum Calcium-transporting ATPase^14^ (Fig. 3). SERCA1a was extracted from native membranes with a 10-fold excess of radiolabeled or cold DDM and both extracts were submitted to two successive Size-Exclusion Chromatographies (SEC, see Methods), the first one being essential to remove the excess of detergent resulting from the solubilization step. We found 159 ± 24 moles [^14^C]-DDM/mole of SERCA1a in the peak corresponding to SERCA1a purified in presence of radiolabeled DDM (upper panel), as previously reported^14^. In parallel we measured 155 ± 27 mol DDM/mol SERCA1a by MALDI-TOF MS from the peak corresponding to the purified SERCA1a in presence of cold DDM (Fig. 3, lower panel), highlighting the accuracy of the present method without the need of using radioactive compounds.

The strategy consisting in coupling SEC and MALDI-TOF MS is therefore promising to quantify the amount of free detergent molecules, and detergent bound to a membrane protein. In the present conditions, it is however necessary to first determine the elution volume of detergent micelles on SEC to avoid any overlap between free micelles and membrane protein-detergent complex on the column that would impair proper determination of membrane protein:detergent ratio (see e.g. the case of LMNG below). In such scenario, a different column may be used such as ion exchange chromatography or immobilized metal affinity chromatography (IMAC) to separate the species.

### Quantification of detergents in complex with BmrA

**Figure 4:**
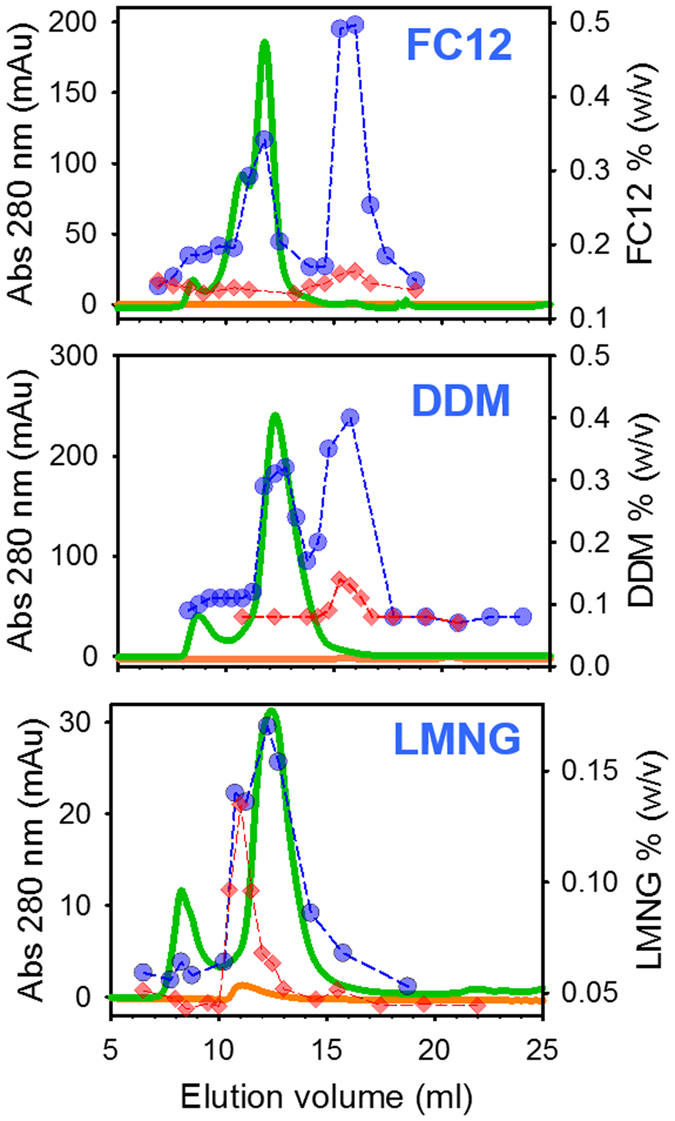
Size Exclusion Chromatography of BmrA in complex with various detergents. BmrA was purified with FC12, DDM and LMNG on IMAC, from which the concentrated pools were concentrated and submitted to SEC. BmrA was probed by its absorbance at 280 nm (green) and detergents quantified by MALDI-TOF MS (blue). The same was done without BmrA (absorbance in orange trace and detergent amounts in red diamonds). Quantifications were done in triplicate on the same experiment.

We tested this strategy on BmrA extracted and purified with FC12, DDM and LMNG^18^,^26^ by IMAC followed by SEC (see Methods and legend of Fig. 4). Detergents were quantified both in the running buffer and in the fractions corresponding to BmrA (pool and/or peak head from the SEC, Fig. 4, Supplementary Figure 4 and Tables 2 and 3).

As shown, FC12, DDM and LMNG co-eluted with BmrA, at 11.7 mL for FC12 and 12.3 mL for DDM and LMNG (green lines), thus corresponding to a mixed membrane protein-detergent population. Such slight difference in elution volumes was also reported for other membrane proteins in complex with different detergents^27^,^28^. Since all these complexes are eluted at about the same volume regardless of the corresponding detergent free micelle size (see below), these data show that in solution, a membrane protein is not embedded into a detergent micelle but forms an independent “membrane protein-detergent” complex. Such observation follows up similar conclusion previously reached by SEC coupled to analytical ultracentrifugation^1^,^29^ and by single crystals neutron diffraction^30^,^31^. Regarding FC12, we recently established that the shoulder visible at 10.5 ml corresponds to OmpF, a membrane protein of the outer membrane of *E. coli* that FC12 extracts efficiently, in contrast to DDM or LMNG^26^, and that we crystallized by happenstance as a contaminant of BmrA^32^.

Quantification of each detergent co-eluting after SEC with the dimer of BmrA gave an amount of 589, 412 and 157 molecules of FC12, DDM and LMNG, respectively (Table 1). These numbers are constant throughout the whole elution volume of the elution peak of BmrA on the SEC (Supplementary Table 2). As a last validation proof, the amount of DDM bound to BmrA quantified by the present method is close to the 380 ± 150 mol/mol BmrA previously reported by using ^14^C-DDM^33^.

### Detection of free micelles by SEC and the atypical behavior of LMNG

In the conditions used in Fig. 4, free FC12 micelles eluted at a peak centered at 15.7 mL (upper panel, dashed lines), corresponding to a MW of 38 kDa, ranging between 16 and 65 kDa (see Supplementary Figure 4 for SEC calibration). Free DDM micelles eluted in a peak centered at 15 ml (center panel, dashed lines), corresponding to a MW of 53 kDa, ranging between 20 to 95 kDa, in agreement with previous reports^34^,^35^. LMNG is a new detergent that was successfully used to crystallize the β2 adrenergic receptor (β2AR)^36^. To the best of our knowledge, we report here for the first time its micelle properties. We observed that free LMNG micelles elute in a peak centered on 11 mL (red diamonds in Fig. 4, lower panel), which in addition displays a delayed elution lasting until 13 mL. A similar type of delayed elution was also observed for LDAO (Lauryldimethylamine N-oxide) over a silica-bed SEC for which the authors concluded on an interaction of micelles with the resin^34^. It may be also the case here with the dextran polymer of the SEC resin. Free LMNG micelle elution peak fits with a size of 393 kDa, ranging between 235 and 622 kDa. A so high range of size remains unusual. LMNG may possibly form unconventional micelles with particular mobility properties, maybe resulting from a non-spherical, e.g. tubular, shape.

Intriguingly, we observed that in these experiments, when BmrA was concentrated prior to the SEC, both FC12 and DDM co-concentrated as free micelles although the concentrator pore size used (routinely 50 kDa, sometimes 100 kDa) was large enough to prevent free detergent accumulation. This suggested that the presence of the membrane protein increases the amount of detergent in the sample. This is clearly visible when comparing blue (with BmrA) and red (without BmrA) symbols in the upper and center panels of Fig. 4 by the large increase of the amplitude peak for free micelles.

### Quantification of bound detergents in complex with several membrane proteins

We expanded this approach to measure the amount of different detergents used to purify a panel of membrane proteins displaying various α-helical or β-barrel topologies, 6 to 12 TMS, mono-, di- and trimeric states (Table 1). We quantified the amount of bound detergents to these proteins over IMAC, SEC or desalting steps. The smallest protein, hAAC1, bound 157–174 FC12 molecules followed by the 7 TMS hP2Y1r with 223 DDM molecules. The 12 TMS LacY, BmrA and BmrCD bound 383, and 328–399 and 444–459 DDM molecules, respectively. Note that the amount of DDM bound to LacY found here is significantly higher than that reported earlier, [142 ± 10 mol DDM + 31 ± 4 mol lipids]/mol LacY^28^ or 121 mol DDM/mol LacY^37^. We do not have a complete explanation about the discrepancy but we could suggest a more important delipidation of the protein in our case, when following the purification protocol used for its crystallization^38^. OprM, the single protein displaying a β-barrel topology, behaves in between with 300 DDM molecules or 364 OG molecules. SERCA1a was notably lower, with only 155 DDM molecules bound. Further experiments are required to understand this difference, maybe due to specific properties such as a much more compact transmembrane domain, a tilted membrane insertion, or the type of membrane (reticulum) in which SERCA1a is natively inserted.

### Detergent – Membrane proteins relationships

**Figure 5:**
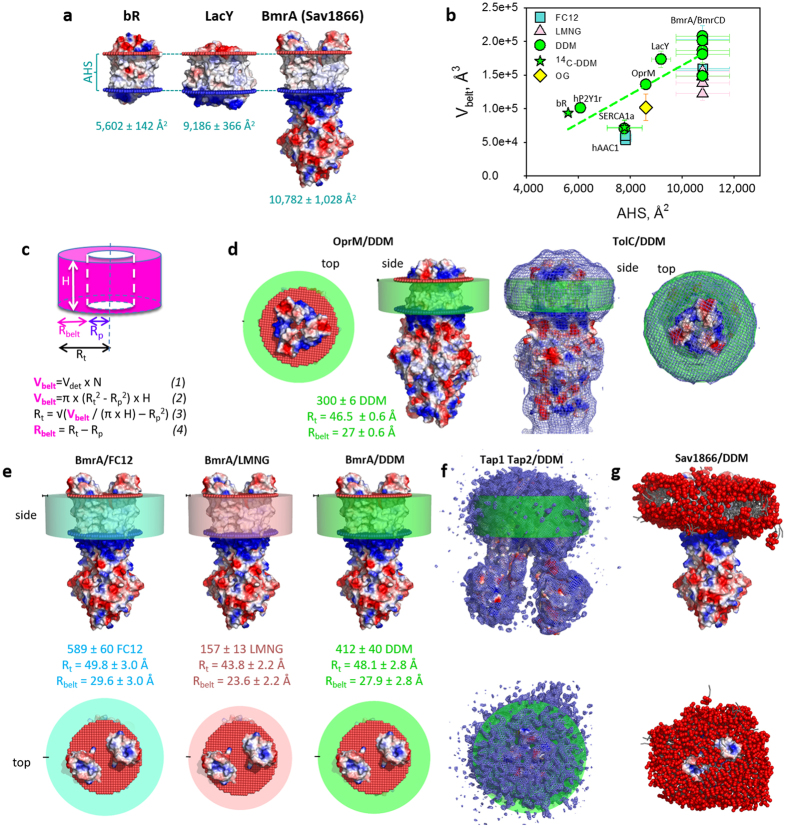
Quantifying and modeling the detergent belt surrounding the hydrophobic region of a membrane protein. (**a**) AHS of bR, LacY and Sav1866 (used as BmrA model) are calculated as described in Methods (see also Supplementary Table 3 and Supplementary Figure 5). (**b**) Detergent belt volume (V_belt_) as a function of AHS. Stars are the ^14^C-DDM bound to SERCA1a (this study) and to bR^1^. (**c**) Belt detergent modelling. V_belt_ is calculated with equations (1,2,3,4) detailed in Methods. (**d**) Top and side views of the OprM-DDM complex. Left: OprM (electrostatic surface), viewed on top from outside the plasma membrane (red dots) and on the side. The calculated DDM corona is in green. Right: side and top views of the cryo-EM map of TolC (16 Å)^53^, close homolog of OprM, displayed in blue mesh, superposed on the OprM-DDM complex model. (**e**) Top and side views of Sav1866-based BmrA model in complex with FC12 (blue), LMNG (salmon) and DDM (green). (**f**) Side and top views of the cryo-EM map of the TAP1/TAP2 ABC transporter (6.5 Å)^41^. (**g**) Snapshot of DDM belt around Sav1866 from a coarse-grained MD simulation. The protein is displayed in electrostatic surface with the detergent in red for the head groups and grey for the alkyl chains.

We then examined the relationships between the detergent bound in such complexes and the membrane domain of the proteins investigated (Fig. 5). On one hand, we considered the volume V_belt_ occupied by the detergent around the membrane domain which can be approximated by equation (1). V_belt_ corresponds to the sum of the volume of each bound detergent molecule V_Det_, Vbelt = N*V_Det_, where N is the number of bound detergent molecules and V_Det_ calculated as described in Methods and Supplementary Table 4. On the other hand, we measured the area of the membrane domain accessible to detergents (accessible hydrophobic surface, AHS) for each membrane protein of the panel, by using either their 3D-structure (SERCA1a, LacY, OprM, hP2Y1r, bR), or those of close crystallized homologs, e.g. hAAC1 and all the structures of ABC exporters available for BmrA and BmrCD (Supplementary Table 3). To do this, we estimated the thickness of the membrane domain and then calculated the accessible hydrophobic area (AHS) (see Methods, Supplementary Table 3, Fig. 5, Supplementary Figure 5).

Depending on the protein, we noticed that the variation of AHS with respect to the conformation of the protein resolved in the corresponding PDB file, was either limited, e.g. 1.5–4% (OprM, bR, hAAC1, hP2Y1r, LacY), or rather large, e.g. 8.7–9.5% (SERCA1a, BmrA).

The resulting plot, V_belt_ = f(AHS) (Fig. 5b) shows that the points are rather fairly linearly distributed, at least for the set of proteins tested here that ranges between 6 and 12 TMS. Fit of the DDM data set (green circles and stars) led to the relation V_belt_(DDM) = (21.7 ± 4.8)*AHS – 52,170 ± 43,945. As V_belt_ (DDM) = N*V_DDM_, we can approximate the number N of bound DDM molecules to a given membrane protein. The amount of bound DDM to BmrA and BmrCD (SEC peak head) was predicted to be around 400 molecules, which compares well to the experimental measures, 399 and 444 molecules, respectively. Good agreement between prediction and experiment was also observed for OprM, with 296 DDM predicted and 300 measured. For LacY, the value of 324 predicted (with DDM) still remained reasonably close to measured value (383 molecules). The method therefore easily provides a first estimation of the amount of detergent bound to membrane proteins.

### Detergent belt modeling

With the values obtained above, we propose a simple method to visualize the detergent belt around a given membrane protein and to approximate its diameter. As previously visualized by neutron diffraction^31^,^39^,^40^, a detergent belt can be approximated to a hollow cylinder (Fig. 5c). The cylinder volume corresponds to V_belt_ and its height H corresponds to the thickness of the membrane as measured above. With these parameters, the radius of whole cylinder R_t_ can be deduced from equations (1, 2, 3, 4). The central hole of the cylinder corresponds to the volume occupied by the membrane domain, also assumed for simplification to adopt a circular shape. Its radius R_p_ can be estimated as detailed in Fig. 5d for the protein OprM, for which we calculated that R_t_ = 46.5 Å and R_belt_(DDM) = 27 Å. Such estimation of the corona diameter seems rather accurate as judged by the quite good superposition of the DDM corona calculated here with the DDM shell experimentally resolved by cryo-electron microscopy (cryo-EM) around TolC, the *E. coli* homolog of OprM displayed on the right handside of panel b. Other detergent corona models are displayed in Supplementary Figure 6. The same superposition done with the DDM corona surrounding BmrA and the recent cryo-EM map of the ABC exporter TAP1-TAP2^41^ (left model of BmrA-DDM in panel e compared to TAP1 TAP2-DDM in panel f of Fig. 5) was also very similar.

Another illustration is given with BmrA in complex with FC12, LMNG or DDM (Fig. 5e), and OprM in complex with DDM or OG (Supplementary Figure 6). The corresponding models show that for BmrA, the R_belt_ of FC12, LMNG and DDM is estimated to 30, 24 and 28 Å, respectively, suggesting that LMNG belt may generate a smaller belt than DDM or FC12; for OprM, the estimated R_belt_ of DDM and OG are of 27 and 21.2 Å, respectively, again suggesting that OG allows a better packing than DDM around the membrane domain. Note that these conclusions differ from those raised with the tetrameric water channel in complex either with LMNG (R_belt_ of 15 Å) or DDM (R_belt_ of 13 Å) when observed by cryo-EM at low resolution (24–25 Å)^42^. A possible explanation for this difference may come from the observation also by cryo-EM of the LMNG-TRPV2 tetramer complex, which, at a higher resolution (13.5 Å) showed that the belt thickness of LMNG around the protein is irregular (15–20 Å)^43^. Such information of the belt thickness, still unpredictable, is crucial for membrane protein crystallization as the crystal quality based on protein-protein contacts is hampered by the bulk of detergents that tends to limit these contacts, and thus a smaller corona will allow better protein-protein interactions. This was exemplified with the reaction center of *Rhodopseudomonas viridis*^44^ for which the success of crystallization was the addition of heptanetriol. It was later shown by neutron diffraction that the size of the detergent belt matches the crystal packing, and by neutron scattering in solution that the size of the detergent micelles is shrunk when heptanetriol is added^45^. This is particularly critical for integral membrane proteins (e.g. LacY in Supplementary Figure S6) and may explain why the exchange of DDM by LMNG dramatically improved the resolution of the β2 adrenergic receptor–Gs protein complex X-ray structure (in addition to a possible stabilization effect)^36^. Our method therefore simply and accurately helps selecting the detergent leading to the lowest steric hindrance of the detergent corona to increase the probability of crystallization of the membrane protein-detergent complex.

Finally, a more precise although time consuming dynamic model of the detergent-protein complex can be obtained by coarse-grained MD simulation (see Methods). This is illustrated by Sav1866 complexed to 400 DDM molecules (Fig. 5g). The detergent forms a belt around the transmembrane moiety of the protein, stable in simulations on the microsecond time scale. Again, the size and position of the DDM belt matches well the estimates (green belt on the left in panel e), but its shape is irregular and fluctuates during the simulation (Supplementary movie). Interestingly, the simulation shows that small, dynamic hydrophobic patches (detergent packing defects) are often present on the surface of the detergent belt, illustrating its high fluidity.

## Discussion

Detergent quantification has hampered membrane protein biochemical studies due to the lack of simple and/or high-throughput techniques to measure the amount of detergent present around membrane proteins in any condition. SEC is a method often used for detergent quantification and which remain handy in labs having only access to SEC. Nevertheless, it has its drawbacks, the main one arising if the micelle size is close to the detergent-protein complex; in addition, it cannot be used to quantify detergent traces upon lipid reconstitution of solubilized membrane proteins. We developed a method using MALDI-TOF MS, simple to implement in laboratories because only the addition of a standard molecule is required for quantification. Interestingly, this technique can be applied to any step of membrane protein preparation, and does not require to specifically process the sample. This method can quantify virtually any detergent, without requiring radioactivity or specific chemical synthesis. Hence, this method could even be applied to the future generations of detergents, and more generally surfactants that will be synthesized.

We have validated this method by a variety of criteria in agreement with previous studies^14^,^33^,^34^,^35^. The level of detection of each detergent will vary depending on its chemical nature. DDM and FC12 can be accurately measured in solution until 0.002%, well below their CMC, and these two detergents can still be detected with a further 10- or 50-fold dilution, respectively. This highly sensitive method allows very low levels of detection e.g. in dialysis experiment. Moreover, since detergents have different masses, this technique is among the few to be able to detect mixtures of detergents in membrane protein preparations. We showed the detection of a DDM/OG mixture over a wide range of concentrations, which will be useful when exchanging detergents or using mixture of detergents. For instance, in the case of the *E. coli* glycerol-3-phosphate transporter, both the exchange of detergents and the use of detergent mixture were necessary to reach 3.3 Å resolution in X-ray crystallography^46^.

Several conclusions can be drawn from our study.

- The amount of detergent bound to membrane proteins varies significantly depending on the size of the membrane protein, and the type of detergent used. While this conclusion seems intuitive, it had seldom been extensively investigated, and our study brings a complete report of detergent quantities around several membrane proteins belonging to various families.
- Purification of BmrA over IMAC followed by SEC using 3 different detergents reveals that the amount of each detergent around the protein stays constant during the purification. This suggests that membrane proteins recruit a given amount of detergent to shield their hydrophobic area that remains constant during the whole purification process. Consistent with this idea, Hauer and colleagues developed an elegant approach to improve the quality of cryo-EM samples. Based on the fact that the exchange rate of LMNG in solution and LMNG surrounding the membrane protein is very slow, they could remove almost all the detergent in solution before freezing the particles for Cryo-EM analysis^47^.
-  The 3 detergents used to purify BmrA have 12 carbons on their aliphatic chains but display different aggregation numbers, MW and micelle shapes, prolate for FC12 and oblate for DDM^35^. Despite the fact that the FC12 micelle size is smaller than that of DDM (38 vs 53 kDa, 54 vs 78–149 monomers), the amount of detergent molecules bound to BmrA is in a similar range. LMNG micelles are large, reported here for the first time to be of 393 kDa (about 400 monomers), yet the amount of LMNG molecules bound to BmrA is about half that of DDM, presumably because the chemical structure of LMNG resembles a twin-like DDM. These observations suggest that membrane proteins are not embedded in a micelle, but rather sequester a minimal amount of detergent sufficient to shield their hydrophobic area.
- Quantification of various detergents with membrane proteins varying in size, shape, type and size of membrane domains shows that the amount of detergent shielding the membrane domains approximatively obeys to a linear relationship between the volume of bound detergent and the accessible hydrophobic surface of these membrane proteins for a given detergent. We show here that such information can be easily obtained, with a rather decent accuracy with DDM, which will be improved with more results.
- The detergent belt can be easily modeled by approximating it to a hollow cylinder. The use of the cylinder model allows to visualize rapidly the difference in detergent belt size, e.g. as we observed when comparing FC12, LMNG and DDM bound to BmrA or DDM and OG bound to OprM. Such a parameter has been early found critical for favoring protein-protein contacts during crystallization assays^30^,^31^, and is another illustration of the power of our method, being able to quantify any detergent bound to any membrane protein.

The use of this simple method may help to unlock the structure resolution of refractory membrane proteins. We expect the MALDI-TOF MS detergent quantification to become a method of reference to investigate membrane protein-detergent complexes thereby improving knowledge in the field of functional and structural biology of membrane proteins.

## Methods

FC12^H^, FC12^D^, DDM^H^, DDM^D^, OG^H^, OG^D^, LMNG, DMNG, CHAPS and CHAPSO were from Anatrace. C4C10 and C4C12 were from CALIXAR. BSA (Fraction V, MW = 65.5 kDa) and lysozyme were from Euromedex. Buffers and solutes, ovalbumin and dextran (80 kDa MW standard) were from Sigma-Aldrich.

### MALDI-TOF MS

Mass spectrometry was performed using a Voyager-DE Pro MALDI-TOF Mass Spectrometer (AB Sciex, Framingham, MA) equipped with a nitrogen UV laser (λ = 337 nm, 3 ns pulse). The instrument was operated in the positive reflectron mode (mass accuracy: 0.008%) with an accelerating potential of 5 kV. All volumes were weighted on a precision scale to maximize accuracy and to compensate for pipetting errors. Detergents (measured and standard) are mixed typically in 50 μL, then 1 μL of the mixture is added of 9 μL of its optimized desorption-helper matrix solution: 10 g DHBA/L water with DDM, OG and CHAPS; 10 g CHCA/L 50:50 acetonitrile:water for FC12 and C4C10/12; 10 g THAP/L 30:70 acetonitrile:water for D/L-MNG; 10 g 9AA/L 50:50 acetone:methanol for cholate and deoxycholate. Except for FC12 and cholate/deoxycholate, 1 μL of 10 g NaI/L acetone was added to the detergent/matrix mixture to produce MNa^+^ cations. However, in the case of Lauryl- and Decyl-MNGs, the 2′,4′,6′-trihydroxyacetophenone (THAP) was found more suitable because of the need of lower energy transferred during the ionization process leading to a more stable signal. The 9-aminoacridine (9AA) was found to efficiently desorb the bile derivatives cholate and deoxycholate^48^.

One microliter of the final solution was laid on the MALDI target and air-dried before analysis. For each trial, mass spectra were obtained by accumulation of 3 series of 300 laser shots, each acquired in 3 distinct areas of the dried mixture. Samples containing high concentrations of imidazole were diluted 10 times before addition of the matrix for favoring crystallization of the mixture. For the analysis of samples containing D/L-MNG, 1 μL of tetrahydrofuran was added onto the dried spot to homogenize it, and air-dried again before data acquisition. Standard curves were fitted with SigmaPlot V12.5.

The optimum acceleration voltage was set to 5,000 V for all detergents tested. This voltage, lower than those used in classical MALDI-TOF MS, gives less energy in the ionization process, leading to stable ionization conditions. As an example, the calibration curve for DDM clearly became non-linear as the voltage increased from 5,000 to 25,000 kV (Supplementary Figure 2).

Detergents desorbed quite differently (Fig. 1b), with a high abundance for deuterated/protonated (H/D) FC12, OG and CHAPS/CHAPSO (2,000 to 20,000 counts) and rather low for (H/D) DDM or L/D-MNG (200 to 2,000 counts). Each triplicate series, displayed highly variable abundances (Fig. 1c). However, the relative laser desorption efficacies of the measured and standard detergents remained remarkably similar (Fig. 1d), resulting in measured to standard ratio close to the theoretical value. In most cases, the linear fit led to experimental slopes of 0.1, which indicate that the two detergents desorb similarly, whereas we reproducibly obtained 0.143 for LMNG, suggesting that it desorbs less efficiently than DMNG, but still proportionally.

### Automation

For further high-throughput analysis, we automated the MALDI-TOF MS acquisition process, for treating up to 100 samples in a row, by using the automatic tool embedded in the Voyager 5.1 (Sciex) software. We tested the set up with DDM and FC12. The acceptance and adjustment threshold of the software were based on an interval of signal intensities such that the signals of the molecular ions 533/558 (DDM + Na^+^) or 352/390 (FC12 + H^+^) fall below a signal-to-noise ratio of 10. We got these settings by choosing an automatic set up of (i) the laser beam intensity value, (ii) the laser beam displacement over the spot, and (iii) the accumulation of nine series of 100 laser shots. In these conditions, the acceptance criteria were found within an interval of 1,000–30,000 counts for a m/z of 533 and 10,000–40,000 counts for a m/z of 352.

### Repeatability and reproducibility

We checked the repeatability and reproducibility of the method for FC12 and DDM by measuring 3 independent experiments respectively on the same day and over three distinct days. The results satisfactorily showed an intra-day average coefficient of variation (CV) of 0.5–12.8% and 0.5–3.8%, and an inter-day CV of 9–18% and 4.0–6.0%, for FC12 and DDM respectively (Table 1).

### Accessible Hydrophobic Surface (AHS) of the membrane domain calculation

Membrane size limits were determined by using the Orientation of Proteins in Membranes online server^49^, http://opm.phar.umich.edu/server.php 56. PDB entries were submitted to the server which placed the proteins in the same orientation and allowed to depict them in a same scale and orientation (Supplementary Figure 5). The software Naccess, V2.1.1^50^, with a probe radius of 1.5 Å (C-C bond length, corresponding to the alkyl chain of detergents) was used to determine total AHS, of which membrane-AHS was extracted using a home-made software, measuring the accessible surface area only contained within the membranous limits determined by the OPM server above. All the PDB entries available for the type of membrane protein tested on this study were subjected to this analysis. Given all the different conformations sampled by the PDB entries, the error on the membrane-AHS calculation was less than 10%.

### Cylinder-shape modeling of detergent belt surrounding membrane proteins

The volume of the detergent belt, V_belt_ can be approximated to the sum of the volume V_det_ occupied by each detergent molecule,


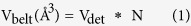


V_det_ is calculated for a given detergent by using the program VOIDOO^51^ (http://xray.bmc.uu.se/usf/voidoo.html) (Supplementary Table 4). The number of bound detergent molecules, N, is determined experimentally by MALDI-TOF MS.

V_belt_ can also be approximated to a hollow cylinder surrounding the membrane region of the protein, the inner volume being occupied by the protein (scheme in Fig. 5c). It can be determined by equation (2) below:


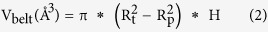


where R_t_ (Å) is the radius of the whole cylinder including protein and detergent, R_p_ (Å) being the radius of the protein cylinder and H (Å) the thickness of the membrane bilayer. R_p_, is obtained by averaging 5–10 distances throughout the membrane domain of a given protein at the inner and outer membrane boundaries, parallel to the membrane plane, using the coordinate files of a given protein for which the 3D-structure is known or that of close homologs and a software for visualizing the 3D structures such as SwissPDBviewer^52^ (v4.1) or PyMOL (The PyMOL Molecular Graphics System, Version 1.8 Schrödinger, LLC.). H is determined by submitting the same coordinates to the OPM server^49^ (http://opm.phar.umich.edu/server.php) that determines the membrane boundaries according to electrostatic potentials.

R_t_ can be deduced from equation (2) as follows:


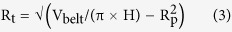


Finally, the radius of the detergent belt, R_b_, can be deduced with the equation (4) below:


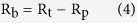


The detergent occupied volume is then displayed as a cylinder of radius R_t_ and height H around the membrane protein using Pymol.

## Additional Information

**How to cite this article:** Chaptal, V. *et al*. Quantification of Detergents Complexed with Membrane Proteins. *Sci. Rep.* **7**, 41751; doi: 10.1038/srep41751 (2017).

**Publisher's note:** Springer Nature remains neutral with regard to jurisdictional claims in published maps and institutional affiliations.

## Supplementary Material

Supplementary Information

Supplementary Video 1

## Figures and Tables

**Table 1:** Quantification of bound detergents to membrane proteins.

Proteins		Det.	step	[Prot] (μM)	Cor.[Det] (mM)	Det./Prot. (mol/mol)
SERCA1a (10 TMS)	Monomer 994 res., 109.5 kDa	DDM	SEC	6.03	0.934	155 ± 27
BmrA (2 × 6 TMS)	Homodimer 2 × 590 res. 130 kDa	FC12	IMAC	12.3	5.7	463 ± 18
			IMAC	2.7	1.2	457 ± 17
			SEC	8.4	4.9	589 ± 60
		DDM	IMAC	7.5	2.5	328 ± 8
			SEC (peak average)	9.5	3.7	412 ± 40
			SEC	11.9	1.6	399 ± 30
		LMNG	IMAC (pool)	2.5	0.5	176 ± 28
			IMAC	4.4	0.8	138 ± 11
			SEC (peak average)	4.4	0.9	157 ± 13
			SEC	5.9	0.9	167 ± 10
BmrCD (6 + 6 TMS)	Heterodimer 585 + 673 res. 138.4 kDa	DDM	IMAC	34.5	15.9	459 ± 12
			SEC	2.5	1.1	444 ± 12
LacY (12 TMS)	Monomer 417 res., 47.2 kDa	DDM	SEC	12.2	4.7	383 ± 27
hAAC1 (6 TMS)	Monomer 298 res., 33 KDa	FC12	SEC	27.0	4.3	157 ± 22 174 ± 10
OprM (β barrel)	Trimer 3 × 474 res. 156.3 kDa	DDM	SEC	38.0	11.4	300 ± 6
		OG	SEC	4.84	1.77	364 ± 70
hP2Y1r (7 TMS)	Monomer 268 res., 30.3 kDa	DDM	Desalting	27.8	6.2	223 ± 6

SERCA1a is the Ca^2+^-transporting ATPase from rabbit, BmrA and BmrCD^54^ are ABC transporters from *Bacillus subtilis*, LacY^55^ is the lactose transporter of *E. coli*, hAAC1^56^ is the human ADP/ATP carrier, OprM^57^ is a component of the MexA-B/OprM drug efflux system of *Pseudomonas æroginosa*, hP2Y1r^58^ is the human purinergic receptor. Proteins were purified by IMAC and SEC as indicated (Methods) and detergents quantified. When not indicated, the SEC or IMAC fraction tested corresponds to the peak head. Quantifications were done in triplicate on the same experiment.
