# Supplementary material for: Quantification of Detergents Complexed with Membrane Proteins
Source: Sci Rep. 2017 Feb 8;7:41751. doi: 10.1038/srep41751 (PMC5297245; doi:10.1038/srep41751)
Supplement: Supplementary Information [file srep41751-s1.pdf]

SUPPLEMENTARY INFORMATION

QUANTIFICATION OF DETERGENTS COMPLEXED WITH MEMBRANE PROTEINS

Vincent Chaptal[1]‡, Frédéric Delolme[2]‡, Arnaud Kilburg[1]‡, Sandrine Magnard[1], Cédric Montigny[3], Martin Picard[4], Charlène Prier[1], Luca Monticelli[1], Olivier Bornert[5], Morgane Agez[6], Stéphanie Ravaud[7], Cédric Orelle[1], Renaud Wagner[5], Anass Jawhari[6], Isabelle Broutin[8], Eva Pebay-Peyroula[7], Jean-Michel Jault[1], H. Ronald Kaback[9], Marc Le Maire[3] & Pierre Falson[1]‡*

[1]Laboratory of Molecular Microbiology and Structural Biochemistry, CNRS UMR 5086, IBCP, Lyon 69367 France,

[2]Protein Science Facility, UMS 3444, IBCP, Lyon 69367 France

[3]Institute for Integrative Biology of the Cell (I2BC), CEA, CNRS, Université Paris-Sud, 91198 Gif-sur-Yvette, France

[4]Laboratoire de Biologie Physico-Chimique des Protéines Membranaires, CNRS UMR 7099, 75005 Paris France

[5]Institut de Recherche de l'Ecole de Biotechnologie de Strasbourg, University of Strasbourg-CNRS, Illkirch, France

[6]CALIXAR, 60 Avenue Rockefeller - 69008 Lyon France,

[7]Institut de Biologie Structurale (IBS), Univ. Grenoble Alpes, CEA, CNRS, 38044 Grenoble, France

[8]Laboratoire de Cristallographie et RMN Biologiques, CNRS UMR 8015, 75006 Paris, France,

[9]Department of Physiology, David Geffen School of Medicine, UCLA, Los Angeles, CA 90095 USA

* pierre.falson@ibcp.fr

‡These authors contributed equally to this work.

a

Detergent	Formula	m/z	Standard	Formula	m/z	Ratio
C4C10	$C_{44}H_{50}O_{10}Na$	761.3 (MNa^+)	C4C12	$C_{46}H_{54}O_{10}Na$	789.3 (MNa^+)	A_{761}/A_{789}
LMNG	$C_{47}H_{88}O_{22}$	1,027.6 (MNa^+)	DMNG	$C_{43}H_{80}O_{22}$	971.6 (MNa^+)	A_{1027}/A_{971}
CHAPS	$C_{32}H_{58}N_2O_7S$	637.4 (MNa^+)	CHAPSO	$C_{32}H_{58}N_2O_8S$	653.4 (MNa^+)	A_{637}/A_{653}
Cholate	$C_{24}H_{40}O_5$	407.3 $(M\text{-}H)^-$	Deoxycholate	$C_{24}H_{40}O_4$	391.3 $(M\text{-}H)^-$	A_{407}/A_{391}

b

Detergent	Formula	m/z	Deuterated standard	m/z (deuterated)	x_{M+1}	x_{M+2}	Ratio
FC12	$C_{17}H_{38}N_1O_4P$	352.3 (M^+)	$FC12^D$	390.3 (M^+)	0.206	0.028	$A_{352}/A_{390real}$
DDM	$C_{24}H_{46}O_{11}$	533.3 (MNa^+)	DDM^D	558.3 (MNa^+)	0.282	0.060	$A_{533}/A_{558real}$
OG	$C_{14}H_{28}O_6$	315.2 (MNa^+)	OG^D	339.2 (MNa^+)	0.166	0.025	$A_{315}/A_{339real}$

c $A(P) = A(P)_{cor} + A(P')_{cor} + A(P'')_{exp}$ (sup1)

$A(P)_{cor} = A(P)_{exp} - A(P'+1)_{cal} - A(P''+2)_{cal}$ (sup2)

$A(P'+1)_{cal} = [A(P')_{exp} - A(P''+1)_{cal}] * x_{M+1}$ (sup2.1)

$A(P''+2)_{cal} = A(P'')_{exp} * x_{M+2}$ (sup2.2)

$A(P')_{cor} = A(P')_{exp} - A(P''+1)_{cal}$ (sup3)

$A(P''+1)_{cal} = A(P'')_{exp} * x_{M+1}$ (sup3.1)

d

Deuterated detergents	Eq.	Experimental equation
FC12	1	$A(390)_{exp} + 0.794 * A(389)_{exp} + 0.808 * A(388)_{exp}$
DDM	2	$A(558)_{exp} + 0.718 * A(557)_{exp} + 0.737 * A(556)_{exp}$
OG	3	$A(339)_{exp} + 0.834 * A(338)_{exp} + 0.837 * A(337)_{exp}$

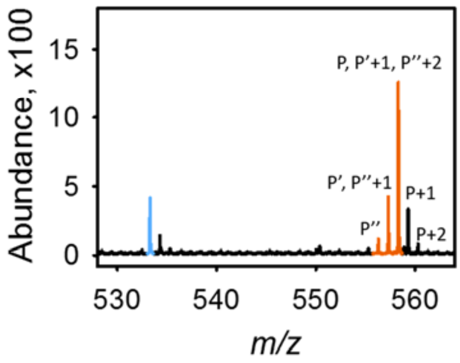

Supplementary Figure 1. MALDI-TOF MS ratio calculation between measured and standard detergent, for deuterated and non-deuterated forms. **a, b**. Tables of detergents and either non-deuterated (a) or deuterated (b) corresponding standards used in the study. M = mass; *A* = absolute abundance of target and standard ions. Relative abundances x_{M+1} and x_{M+2} are those of the second and third peaks of an isotopic pattern normalized to 1. **c**. True abundance of the deuterated standards, *A*(P), taking into account the contribution of (i) the isotopes of C, H, O, N (x_{M+1}, x_{M+2}), (ii) different species of the molecule due to H<->D exchange (P, P', P''). An example is given in the spectrum on the right for deuterated DDM. **d**. Experimental equations for each deuterated standard detergent.

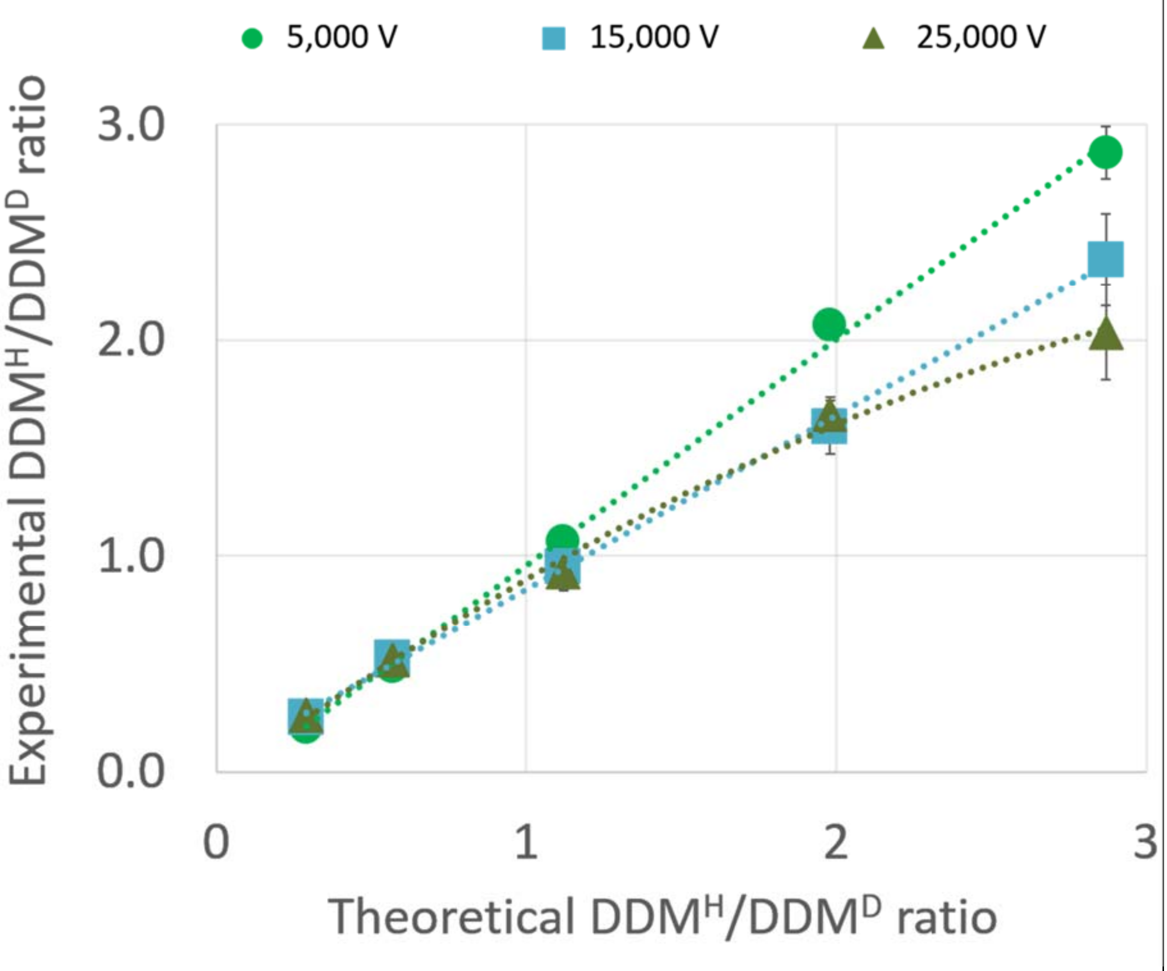

Supplementary Figure 2. Calibration curves at 3 accelerating voltages for DDM assayed at 0, 0.03, 0.1 and 0.3 % using 0.1 % DDM^D as standard. [DDM^H]/[DDM^D]experimental ratios are plotted in respect to [DDM^H]/[DDM^D] theoretical ratios. The experiment was done in triplicate, fitted with a linear regression with Excel.

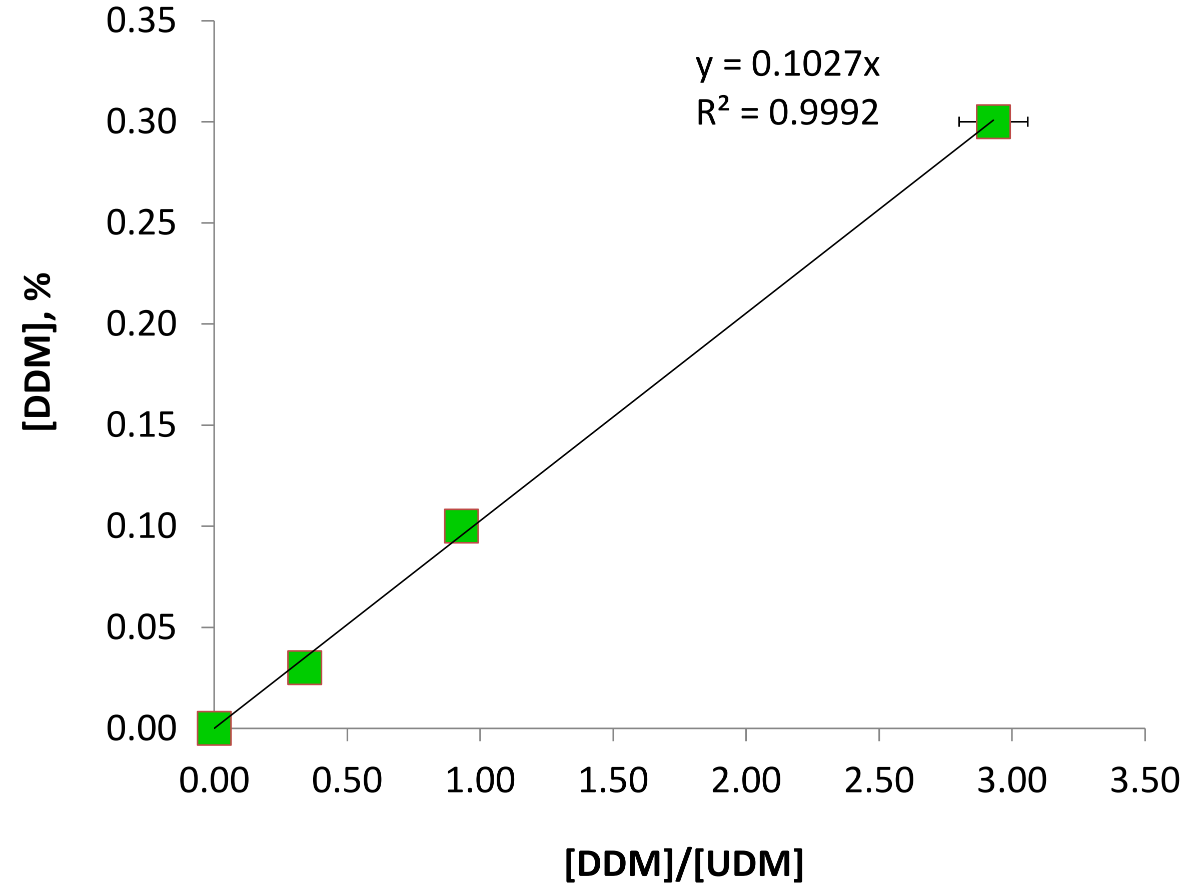

Supplementary Figure 3. DDM quantification with UDM by MALDI-TOF MS. Calibration curve for DDM assayed at 0, 0.03, 0.1 and 0.3 % using 0.1 % UDM as standard. DDM concentration is plotted as the amount of measured DDM (%, w/v) in respect to assayed DDM/UDM abundance ratios. The experiment was done in triplicate, fitted with a linear regression with SigmaPlot V12.5

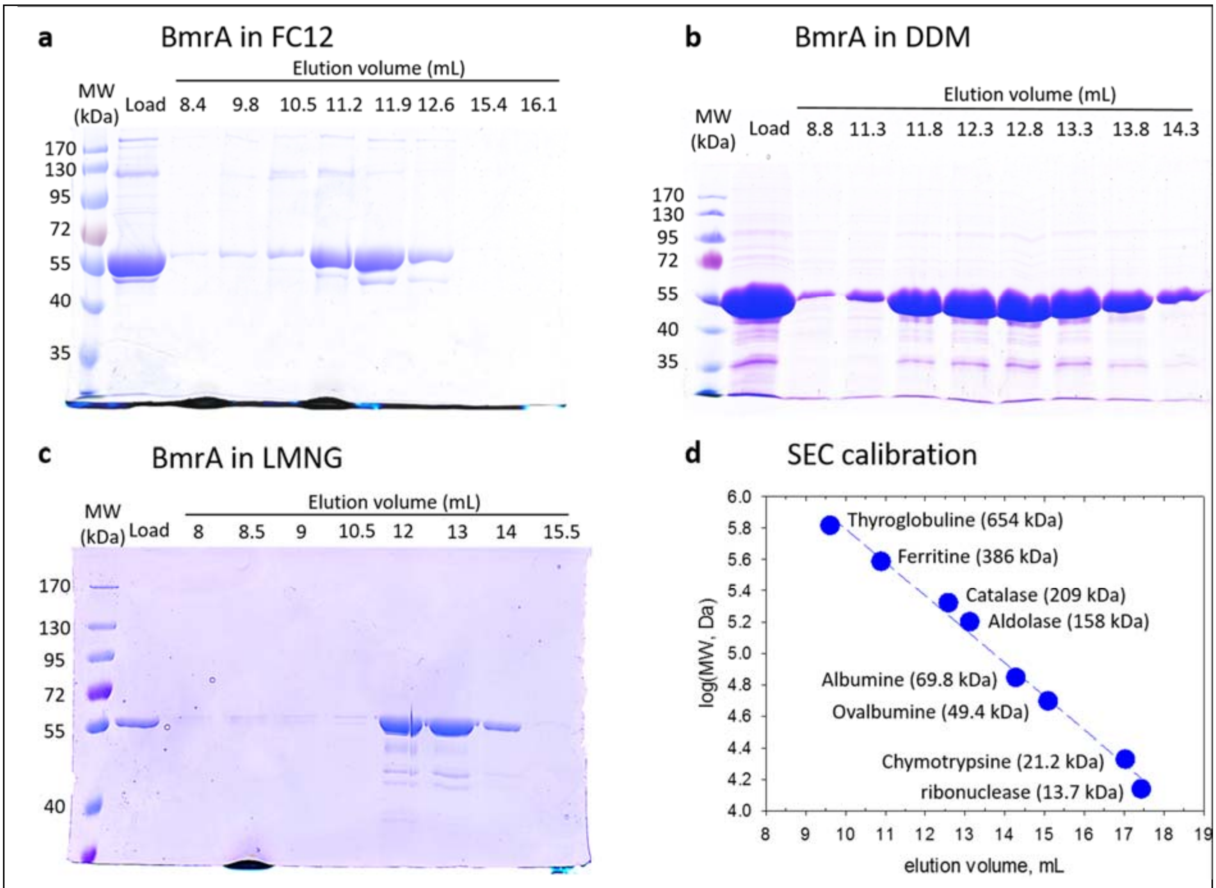

Supplementary Figure 4. SDS-PAGE analysis of the fractions collected after SEC displayed in Figure 3B, and analyzed for detergent quantification in Supplementary Table 2. **a-c**. MW, expressed in kDa on the left of the gel. Load is the sample applied onto the SEC. Fractions are labelled using the elution volume from the SEC. Gels were stained with Coomassie blue. **d**. MW calibration of the Superdex S200 gel filtration column used in the present study. MW standards were injected on the S200 to get their elution volumes. A rough fitting was obtained by plotting the log (MW) as a function of the elution volume, which was found linear as shown between 9 and 18 mL: log (MW, Da) = -0.214 ± 0.008 x Elution volume (mL) + 7.934 ± 0.118; R = 0.995.

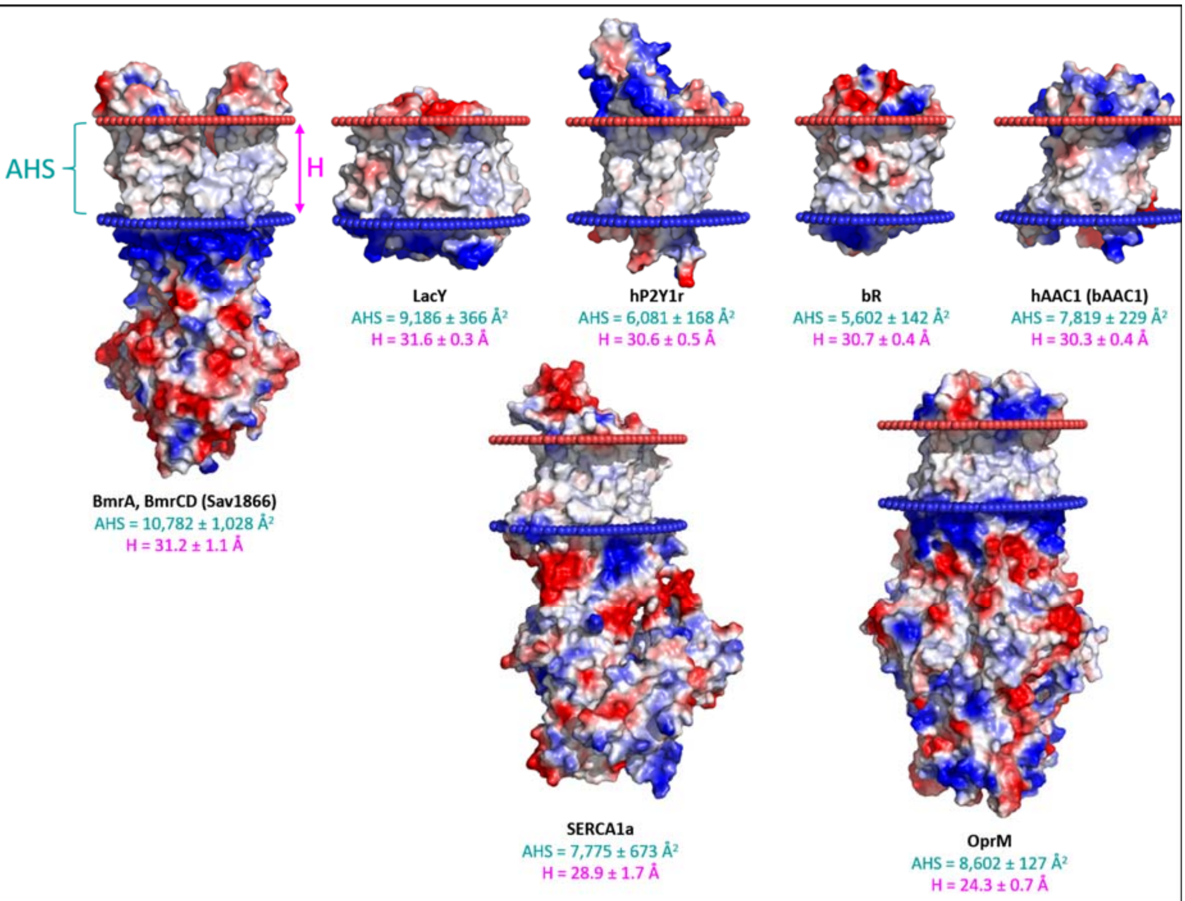

Supplementary Figure 5. AHS and thickness of the transmembrane region (H) of MPs included in the present study. AHS was determined based on the 3D-structure(s) of SERCA1a, LacY, OprM, hP2Y1r, bR, or those of close homologs of hAAC1 (bovine AAC1, PDB code 2C3E), BmrA and BmrCD for which the 3D structure of a homologue has been established (shown: Sav1866, PDB code 2HYD) as displayed in Supplementary Table S3 and detailed in Methods. Membrane limits and AHS were determined as described in Methods.

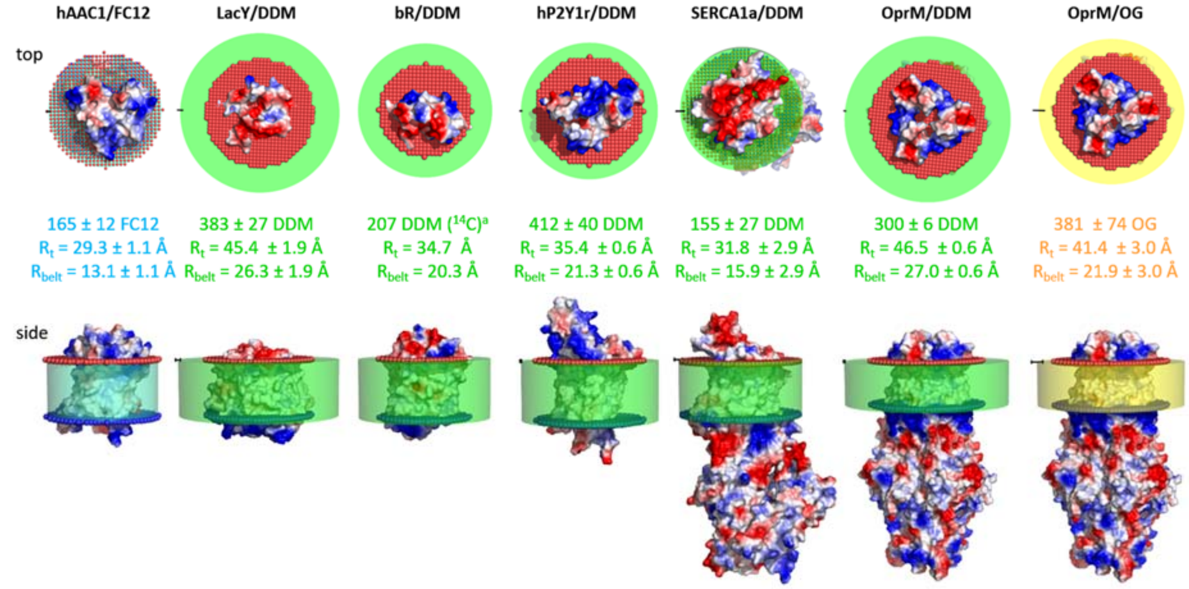

Supplementary Figure 6. Schematized view of the volume occupied by the detergents around the membrane domains of the proteins included in this study. The volume, approximated to a hollow cylinder is calculated as detailed in Figure 4c and Methods. R_t, radius of the membrane domain + detergent belt and R_{belt}, radius of the detergent belt is calculated as detailed in Figure 4c and Methods.

Supplementary Table 1. Intra-day and inter-day CVs for FC12 and DDM detergents. Data are reported as $[FC12^H]/[FC12^D]$ or $[DDM^H]/[DDM^D]$ (theoretical ratios of 0.3-1-3). For intra-day CVs (Day 1, Day2, Day3), data are the mean of 3 experiments done in the same day. For inter-day CVs, data are the mean of Day 1, Day 2 and Day 3.

$FC12^H/FC12^D$	0.3	1	0.3
Intra-day precision			
Day 1			
Mean (n=3)	0.34	0.99	2.77
CV (%)	8.4	3.7	12.8
Day 2			
Mean (n=3)	0.45	1.18	3.38
CV (%)	7.4	3.3	5.0
Day 3			
Mean (n=3)	0.32	1.02	2.86
CV (%)	0.5	3.0	9.5
Inter-day precision			
Mean (n=3)	0.37	1.06	3.0
CV (%)	18.0	9.0	12.0
DDM^H/DDM^D	0.3	1	0.3
Intra-day precision			
Day 1			
Mean (n=3)	0.34	1.15	3.34
CV (%)	3.0	2.2	2.2
Day 2			
Mean (n=3)	0.38	1.04	3.20
CV (%)	1.9	2.4	0.5
Day 3			
Mean (n=3)	0.39	1.09	3.07
CV (%)	3.0	2.3	3.8
Inter-day precision			
Mean (n=3)	0.37	1.09	3.2
CV (%)	6.0	5.0	4.0

Supplementary Table 2. Detergent quantification after SEC displayed in Figure 4. The corrected concentration of each detergent is the concentration measured in the sample minus the buffer detergent concentration. In bold are the fractions taken for the calculation of the average of the peak summarized in Figure 4. For LMNG, since the free micelle is overlapping with the detergent peak, fractions were taken on the opposite fractions of the peak so that the free micelle peak does not interfere with the measurement. The concentration of BmrA correspond to its dimeric (native) form.

Detergent	Elution volume (ml)	[Det]corrected (mM)	[BmrA] (mM)	Ratio Det/BmrA
FC12	9.8	1.54	2.1	739
	10.5	1.46	4.8	306
	11.2	4.22	7.9	532
	11.9	5.62	8.7	646
	12.6	2.43	2.9	833
	13.3	1.71	-	-
	14	0.73	-	-
	14.7	0.76	-	-
DDM	11	0.78	3.38	-
	**11.5**	**4.14**	**9.46**	**438**
	**12.5**	**4.76**	**11.92**	**399**
	**13**	**3.16**	**7.92**	**399**
	**13.5**	**1.68**	**4.46**	**376**
	14	2.31	-	-
	14.5	5.33	-	-
	15.5	6.25	-	-
LMNG	10	0.05	-	-
	10.5	0.79	0.8	-
	11	0.75	2.2	335
	**12**	**1.07**	**5.8**	**184**
	**12.5**	**0.92**	**5.9**	**156**
	**14**	**0.26**	**1.5**	**176**
	15.5	0.09	-	-

Supplementary Table 3. Membrane domain thickness and accessible hydrophobic surface (AHS) calculations of the MPs included in the present study. Both parameters were calculated as described in Methods using all 3D-structures reported for each MP in the Protein Data Bank, except for SERCA1a for which only the 3D-structures in the calcium-bound E1 conformation were considered accordingly to the experimental conditions. For BmrA and BmrCD for which the 3D-structure is not known, we related proteins belonging to the ABC exporter's family.

**Membrane Protein**			**Membrane domain**	
**Family**	**protein**	**PDB code**	**Thickness, Å**	**Accessible Hydro-phobic Surface @1.5 Å, Å²**
**ABC exporters**	Sav1866	2onj	30.4	11,275
		2hyd	31.2	11,373
	MsbA	3b60	32	11,784
	Mouse ABCB1	3g5u	30.4	10,488
		4lsg	31	10,420
		3g60	30.6	10,737
		3g61	30.6	10,973
		4ksb	29.8	11,272
		4ksc	30.8	12,253
		4ksd	30.2	11,327
		4m1m	31	10,341
		4m2s	31.2	10,238
		4m2t	30.8	10,340
	C. elegans ABCB1	4f4c	33	10,558
	ABCB10	4ayt	31	13,055
		4ayx	30.6	12,851
		4ayw	30.4	12,184
		3zdq	30.4	12,758
	TM287/288	3qf4	30.6	9,700
		4q4h	30.8	9,752
		4q4j	30.2	9,779
		4q4a	30.6	9,834
	Atm1 *S. cerevisiae*	4myc	30.8	10,047
		4myh	30.4	9,839
	Na Atm1	4mrn	31.4	9,928
		4mrp	32	9,926
		4mrs	31.6	9,832
		4mrv	31.8	9,880
	McjD	4pl0	31.4	10,089
	CmABCB1	3wmg	35.4	11,414
		3wme	31.2	9,287
		3wmf	33.8	9,811
	PglK	5c78	31.2	11,414
		5c76	32	11,819
	**All**	**Mean**	**31.2 ± 1.1**	**10,782 ± 1,028**

Membrane Protein			Membrane domain	
**Family**	**protein**	**PDB code**	**Thickness, Å**	**Accessible Hydro-phobic Surface @1.5 Å, Å²**
**Efflux Systems**	OprM	1wp1	23.8	8,886
		3d5k (full)	24.8	8,763
	**All**	**Mean**	**24.3 ± 0.7**	**8,602 ± 127**
**ADP/ATP Exchanger**	hAAC1	2c3e	29.8	7,557
		1okc	30.2	7,911
		4c9g	31	7,944
		4c9h	30.6	8,100
		4c9q	30	7,525
		4c9j	30.4	7,876
	**All**	**Mean**	**30.3 ± 0.4**	**7,819 ± 229**
**Major Facilitator**	LacY	2y5y	31.4	9,128
		1pv7	31.2	9,212
		4zyr	32	9,648
		2v8n	31.6	8,757
	**All**	**Mean**	**31.6 ± 0.3**	**9,186 ± 366**
**P-type ATPase**	SERCA E1 conformation	1t5s	27.4	6,914
		1su4	30.2	8,707
		1t5t	27.2	6,852
		1vfp	29.6	7,447
		2c9m	29.6	8,228
		2zbd	32	8,513
		3ba6	30	7,247
		3ar2	29.2	7,815
		3n8g	29.8	7,314
		3w5a	29.8	8,319
		3w5b	29.8	8,639
		4h1w	27	8,207
		4nab	26.8	7,800
		4xou	26.4	6,852
	**All**	**Mean**	**28.9 ± 1.7**	**7,775 ± 673**

Membrane Protein			Membrane domain	
**Family**	**protein**	**PDB code**	**Thickness, Å**	**Accessible Hydro-phobic Surface @1.5 Å, Å²**
**7TM**	hP2Y1r	2ydo	31	6,009
		2ydv	30.8	5,997
		3eml	31.2	6,138
		3pwh	29.6	6,122
		3qak	30.2	5,823
		3rey	30.6	6,177
		3rfm	30.4	6,123
		3uza	30.2	6,138
		3uzc	30.2	6,203
		3vg9	30.2	5,897
		3vga	30.8	5,900
		4eiy	31.6	6,441
	**All**	**Mean**	**30.6 ± 0.5**	**6,081 ± 168**
**7TM Bacteriorhodopsin**	dark	1x0s	31	5,710
	K	1m0k	30.4	5,476
	K	1qkp	30.6	5,615
	L	1ucq	31	5,677
	L	1o0a	31.2	5,523
	M1	1m0m	30.2	5,474
	M1	1p8h	30.4	5,463
	N	1p8u	30.4	5,589
	O	3vi0	31.2	5,895
	**All**	**Mean**	**30.7 ± 0.4**	**5,602 ± 142**

Supplementary Table 4. Detergent properties as given by The Protein Data Bank in Europe (http://www.ebi.ac.uk/pdbe/) and from Anatrace (https://www.anatrace.com/Products/Detergents.aspx). The volume of each detergent is calculated by using the program VOIDOO [1] (http://xray.bmc.uu.se/usf/voidoo.html).

DDM	$C_{24}H_{46}O_{11}$	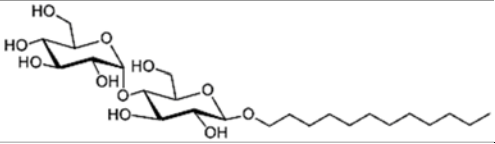
	510.621 g/mol	
	Smiles: CCCCCCCCCCCCOC2OC(CO)C(OC1OC(CO)C(O)C(O)C1O)C(O)C2O	
	Volume : 453 Å^3	
	http ://www.ebi.ac.uk/pdbe-srv/pdbechem/chemicalCompound/show/LMT	
FC12	$C_{17}H_{38}NO_4P$	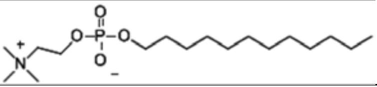
	315.5 g/mol	
	Smiles: CCCCCCCCCCCCOP([O-])(=O)OCC[N+]©©C	
	Volume : 344 Å^3	
	http ://www.anatrace.com/Products/Lipids/FOS-CHOLINE/F308S.aspx	
OG	$C_{14}H_{28}O_6$	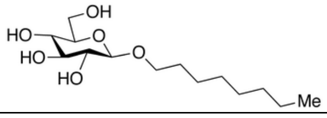
	292.369 g/mol	
	Smiles : CCCCCCCCOC1OC(CO)C(O)C(O)C1O	
	Volume : 267.5 Å^3	
	http ://www.ebi.ac.uk/pdbe-srv/pdbechem/chemicalCompound/show/BOG	
LMNG	$C_{47}H_{88}O_{22}$	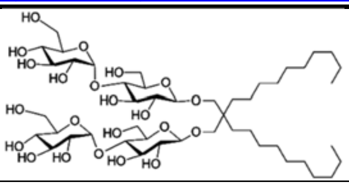
	1,005.19 g/mol	
	Smiles: CCCCCCCCCCC(CCCCCCCCCC)(COC2OC(CO)C(OC1OC(CO)C(O)C(O)C1O)C(O)C2O)COC4OC(CO)C(OC3OC(CO)C(O)C(O)C3O)C(O)C4O	
	Volume : 885.7 Å^3	
	http ://www.anatrace.com/Products/Detergents/NG-CLASS/NG310.aspx	

SUPPLEMENTARY MOVIE

Molecular dynamics simulations of Sav1866 in the presence of 400 DDM molecules. See Methods for details.

SUPPLEMENTARY METHODS

MALDI-TOF MS

Repeatability and reproducibility.

We checked the repeatability and reproducibility of the method for FC12 and DDM by measuring 3 independent experiments respectively on the same day and over three distinct days. The results satisfactorily showed an intra-day average coefficient of variation (CV) of 0.5-12.8% and 0.5-3.8%, and an inter-day CV of 9-18% and 4.0-6.0%, for FC12 and DDM respectively (Table 1).

Ratio calculation between quantified and standard detergents

Under MS ionization, the signature of a molecule P constituted of C, H, O, N corresponds to the distributions of m/z, P, P+1 and P+2, in which m/z of +1 and +2 correspond to the isotopic contribution of each element. This effect is negligible for non-deuterated detergents for which only the intensities of the first peak (P) of the isotopic pattern are considered. For deuterated detergents standards, satellite ions appear at -1 and -2 m/z in the molecular ions region (m/z 388.3 and 389.3 for FC12 (m/z 390.3), 556.3 and 557.3 for DDM (mz 558.3), 337.2 and 338.2 for OG (m/z 339.2) (see Figure 1b). These ions correspond to a 1 or 2 H<->D exchange occurring during the MALDI preparation step and have to be taken into account to generate the standard curves. As detailed in Supplementary Figure S1, considering a compound with a m/z = P and using equations (sup1.1-3), we calculate firstly the true abundance of P’ by eliminating the contributions of the isotopic element of P’’+1, and that of P by eliminating the contributions of P’+1 and P”+2. Then, the true abundance of the deuterated form is calculated by adding the true abundances of P, P’ and P”.

Calculation of the amount of detergent bound around membrane proteins

Detergent quantification gives access to the total amount of detergent in the sample. Therefore, for a sample containing a membrane protein, it corresponds to the detergent complexed with a membrane protein plus any additional detergent present in the buffer. The amount of detergent bound to a membrane protein equals thus the total amount of detergent in the sample (*i.e*. the peak of a SEC column) subtracted from the buffer. Caution should be used in the case of an overlap between the elution volume of free micelles and membrane protein; in such case, detergent quantification should be carried out only on fractions having little or no overlap, or a different chromatographic analysis should be carried out (for example, ion exchange chromatography). The protein concentration is calculated by measuring the OD^{280nm} and using the Beer-Lambert equation. The amount of bound detergent per membrane protein is the ratio of bound detergent over protein concentration.

BmrA reconstitution into liposomes and ATPase activity monitoring

BmrA was purified essentially as described before[2] with the following modifications. Bacteria were lysed by three successive passages through a microfluidizer (18,000 psi) in absence of benzonase, individual antiprotease inhibitors were replaced with tablets of antiprotease complete mini EDTA-free (Roche) and 10 mM ethylenediaminetetraacetic acid (EDTA) was not added following cell lysis. After membrane protein solubilization, the supernatant was loaded with an AKTA system onto a 5 ml pre-packed column (5 ml, Ni^{2+}-nitrilotriacetic acid-agarose) pre-equilibrated with buffer A (50 mM potassium Pi, pH 8.0, 15% glycerol, 100 mM NaCl, 0.05% DDM, 5 mM β-mercaptoethanol) containing 20 mM imidazole. Washing steps were performed with 25 ml of the equilibration buffer, and 90 ml buffer A containing 75 mM imidazole. Elution was performed with a 60 ml buffer A containing an imidazole gradient from 75 mM to 500 mM imidazole. Fractions containing BmrA were collected and twice dialyzed in 500 mL of buffer containing 50 mM Hepes pH 8.0, 10% glycerol, 50 mM NaCl, 0.05% DDM, and 5 mM β-mercaptoethanol. BmrA was then concentrated ~3.5 fold with an Amicon Ultra-15 concentrators (50 kDa cut-off, Millipore) to reach a final concentration of about 0.8 mg/ml, then frozen in liquid nitrogen and kept at -80 °C until use. Reconstitution was performed as previously described[2], except that the initial mixture lipids/detergent was incubated for 45 min instead of one hour. The ATPase activity of BmrA was monitored along the reconstitution process at 37 °C by using an ATP-regenerating system coupled to the disappearance of NADH recorded at 340 nm[3]. Reactions were performed in a final volume of 622 μl of Hepes 50 mM pH 8.0, 10 mM $MgCl_2$, 4 mM PEP, 60 μg/ml pyruvate kinase, 32 μg/ml lactate dehydrogenase, 0.3 mM NADH, 10 mM ATP and were initiated by adding 1 μg of BmrA.

Membrane proteins purifications.

BmrA was produced and purified as described[4,5]. BmrA extracted with 1% FC12 was incubated for 2 hours at 4°C and then centrifuged for 1 hour at 100,000xg, 4°C. The supernatant was applied onto a IMAC (GE Healthcare), reducing the FC12 concentration to 0.3% in the buffer (20 mM Tris-HCl pH 8.0, 100 mM NaCl). The 5-mL elution pool was concentrated to 0.5 mL on a 50-kDa regenerated cellulose

concentrator (Amicon Ultra, Millipore) and injected on Superdex 200 10/300 GL gel filtration chromatography (GE Healthcare) using as mobile phase 20 mM Tris-HCl pH 8.0, 100 mM NaCl, 0.3% FC12, at 0.5 mL/min.

BmrA was also extracted and purified in DDM and LMNG in a similar way, using a detergent concentration of 1% during extraction and 0.05% for DDM and LMNG for the IMAC step. Each IMAC pool was then concentrated as above and applied on Superdex 200 at 0.5 ml/min with a mobile phase of 50 mM Hepes pH 8.0, 100 mM NaCl, added of either 0.02% DDM or 0.05% LMNG. The same protocol was applied without protein to quantify in each case the detergent in the corresponding fractions. In both cases, fractions were collected, absorbance at 280 nm was measured to quantify BmrA.

SERCA1a Ca^{2+}-ATPase. A SERCA-enriched membrane fraction prepared from rabbit[6] was solubilized with DDM and submitted to SEC with DDM or ^{14}C-DDM as radiotracer to quantify DDM[7]. Briefly, 2 mg of solubilized SERCA1a in 20 mg DDM (cold experiment) or 4 mg SERCA in 40 mg DDM (radioactive experiment) was injected on a TSK3,000SW column equilibrated in 20 mM MOPS-Tris pH 7.0, 25 mM NaCl, 1 mM $CaCl_2$, 2.5 mM $MgCl_2$ and 0.05% DDM. The first chromatography was carried out to discard aggregates, lipids and extra amounts of free detergent used for extraction of SERCA1a. Cold and radioactive fractions corresponding to SERCA were pooled, concentrated with YM-100 type Amicon ultrafiltration device and submitted to the same SEC step, recording absorbance at 280 nm on a HP8453 spectrophotometer for quantifying SERCA by using a mass extinction coefficient of 0.95 $L^{-1}.g^{-1}.cm^{-1}$. Final protein concentration was about 0.7 g/L. Samples were flash-frozen in liquid nitrogen and stored at -20°C.

OprM was produced in *E. coli* and purified as previously described[8]. The protein was extracted in OG and purified by IMAC, using a buffer containing 20 mM Tris-HCl pH 8.0, 10 % glycerol, 0.9 % OG for the mobile phase, added of 250 mM imidazole for the elution step. Purification of OprM in DDM was carried out similarly. Membranes were solubilized in 20 mM Tris-HCl pH 8.0, 10% glycerol and 2% DDM, and applied IMAC, reducing the DDM concentration to 0.05% in the mobile phase. In both cases, OprM fractions were concentrated on 100 kDa cut-off (Amicon Ultra) and submitted to a SEC step (Superpose 6) with a buffer containing 20 mM Tris-HCl pH 8.0, 10 % glycerol and 0.9% OG or 0.05% DDM as mobile phase.

hAAC1 was produced in *E. coli* fused to the Maltose-Binding Protein and to a 8xHis tag at the N-terminus[9]. The fusion protein was extracted with 1% FC12, and then submitted to IMAC. The Maltose-binding protein moiety and His tag were removed by thrombin. hAAC1 was then submitted to a SEC step (Superdex 200 10-300) using 50 mM Tris-HCl pH 7.5, 100 mM NaCl, 0.1 % FC12 as mobile phase. Fractions containing hAAC1 were pooled and concentrated 20 times on a 30 kDa Amicon Ultracel. The same volume of buffer was concentrated identically.

BmrCD was extracted and purified using DDM on IMAC and SEC (Superdex 200) as described previously[10].

hP2Y1r was produced in *Pichia pastoris* using the system described previously[11]. The protein was extracted with 1% DDM, purified on IMAC followed by a desalting step using a buffer containing 50 mM Tris-HCl pH 7.4, 0.5 M NaCl, 0.1 % DDM, 0.01 % cholesterol hemisuccinate as mobile phase. The protein was then concentrated 20 times on a 50 kDa cut-off vivaspin (Amicon Ultra). The same volume of buffer was concentrated identically.

Molecular dynamics simulations

We carried out molecular dynamics (MD) simulations of Sav1866 in the presence of 400 DDM molecules. The crystal structure of Sav1866 was downloaded from the PDB (access code 2HYD). The all-atom structure was converted to a coarse-grained representation using the MARTINI force field[12,13]. DDM molecules were placed around the protein in a bilayer-like arrangement, and the protein-detergent complex was assembled as described[14]. The complex was then solvated in a cubic box with lateral size of about 16 nm, containing 30,899 water particles, 462 Na^+ ions and 458 Cl^- ions, yielding an electrically neutral system with approximately 200 mM NaCl concentration. Non-bonded interactions were calculated

within a cut-off of 1.2 nm, using shift functions (from 0 nm for electrostatics and from 0.9 nm for Lennard-Jones) and a dielectric constant of 15, according to the standard MARTINI setup. Simulations were carried out with the GROMACS (v4.5) software[15] using 3D periodic boundary conditions and the NpT ensemble. Pressure and temperature were kept constant (1 bar, 298 K) using the Parrinello-Rahman barostat[16] and the Bussi-Donadio-Parrinello thermostat[17], with time constants of 1 ps and 5 ps respectively. The integration time step was 25 fs and the total simulation time was 1 µs.

REFERENCES

1 Kleywegt, G. J., Zou, J. Y., Kjeldgaard, M. & Jones, T. A. in *Crystallography of Biological Macromolecules* Vol. F *International Tables for Crystallography* (eds M.G. Rossmann & E. Arnold) Ch. 17.1, 353-356, 366-367 (Kluwer Academic Publishers, The Netherlands, 2001).

2 Orelle, C., Dalmas, O., Gros, P., Di Pietro, A. & Jault, J. M. The conserved glutamate residue adjacent to the Walker-B motif is the catalytic base for ATP hydrolysis in the ATP-binding cassette transporter BmrA. *J. Biol. Chem.* **278**, 47002-47008 (2003).

3 Steinfels, E. *et al.* Characterization of YvcC (BmrA), a multidrug ABC transporter constitutively expressed in *Bacillus subtilis*. *Biochemistry* **43**, 7491-7502 (2004).

4 Steinfels, E. *et al.* Highly efficient over-production in *E. coli* of YvcC, a multidrug-like ATP-binding cassette transporter from *Bacillus subtilis*. *Biochim Biophys Acta* **1565**, 1-5 (2002).

5 Matar-Merheb, R. *et al.* Structuring detergents for extracting and stabilizing functional membrane proteins. *PLoS One* **6**, e18036, doi:10.1371/journal.pone.0018036 (2011).

6 Champeil, P. *et al.* Kinetic characterization of the normal and detergent-perturbed reaction cycles of the sarcoplasmic reticulum calcium pump. Rate-limiting step(s) under different conditions. *J. Biol. Chem.* **261**, 16372-16384 (1986).

7 le Maire, M. *et al.* Gel chromatography and analytical ultracentrifugation to determine the extent of detergent binding and aggregation, and Stokes radius of membrane proteins using sarcoplasmic reticulum Ca^{2+}-ATPase as an example. *Nat Protoc* **3**, 1782-1795, doi:10.1038/nprot.2008.177 (2008).

8 Broutin, I. *et al.* Expression, purification, crystallization and preliminary X-ray studies of the outer membrane efflux proteins OprM and OprN from *Pseudomonas aeruginosa*. *Acta Crystallogr Sect F Struct Biol Cryst Commun* **61**, 315-318, doi:10.1107/s1744309105005014 (2005).

9 Ravaud, S. *et al.* Impaired Transport of Nucleotides in a Mitochondrial Carrier Explains Severe Human Genetic Diseases. *ACS Chemical Biology* **7**, 1164-1169, doi:10.1021/cb300012j (2012).

10 Galian, C. *et al.* Optimized purification of a heterodimeric ABC transporter in a highly stable form amenable to 2-D crystallization. *PLoS One* **6**, e19677, doi:10.1371/journal.pone.0019677 (2011).

11 Andre, N. *et al.* Enhancing functional production of G protein-coupled receptors in *Pichia pastoris* to levels required for structural studies via a single expression screen. *Protein Sci* **15**, 1115-1126, doi:10.1110/ps.062098206 (2006).

12 Marrink, S. J., Risselada, H. J., Yefimov, S., Tieleman, D. P. & de Vries, A. H. The MARTINI force field: Coarse grained model for biomolecular simulations. *Journal of Physical Chemistry B* **111**, 7812-7824, doi:10.1021/jp071097f (2007).

13 Monticelli, L. *et al.* The MARTINI coarse-grained force field: Extension to proteins. *Journal of Chemical Theory and Computation* **4**, 819-834, doi:10.1021/ct700324x (2008).

14 Javanainen, M. Universal Method for Embedding Proteins into Complex Lipid Bilayers for Molecular Dynamics Simulations. *Journal of Chemical Theory and Computation* **10**, 2577-2582, doi:10.1021/ct500046e (2014).

15 Hess, B., Kutzner, C., van der Spoel, D. & Lindahl, E. GROMACS 4: Algorithms for highly efficient, load-balanced, and scalable molecular simulation. *Journal of Chemical Theory and Computation* **4**, 435-447, doi:10.1021/ct700301q (2008).
16 Parrinello, M. & Rahman, A. Polymorphic Transitions in Single Crystals - a New Molecular Dynamics Method. *Journal of Applied Physics* **52**, 7182-7190 (1981).
17 Bussi, G., Donadio, D. & Parrinello, M. Canonical sampling through velocity rescaling. *Journal of Chemical Physics* **126**, 014101, doi:10.1063/1.2408420 (2007).
